# Influencing factors of marathon runners’ sport risk behavior: a mixed method study based on social cognitive theory

**DOI:** 10.3389/fpsyg.2025.1761422

**Published:** 2026-01-16

**Authors:** Haisheng Shen, Zihui Ma, Hongying Wang

**Affiliations:** 1 School of Physical Education, Shanghai University of Sport, Shanghai, China; 2 School of Athletic Performance, Shanghai University of Sport, Shanghai, China

**Keywords:** influence factor, marathon runners, mixed method, social cognitive theory, sport risk behavior

## Abstract

**Introduction:**

Preventing health risks for marathon runners is of great significance for the sustainable development of marathon competitions. This study is based on the perspective of “prevention and control of human unsafe behavior” in system security theory, using social cognitive theory as the theoretical framework. By using a mixed method of triangulation (qualitative research + quantitative verification + literature support), this study explores the influencing factors of marathon runners’ sport risk behavior.

**Method:**

This study first conducted interviews with 21 Chinese marathon runners to summarize potential influencing factors through thematic analysis. Subsequently, 514 quantitative data were collected from marathon events in China, and Spearman correlation analysis was employed to verify the relationship between these influencing factors and the level of sport risk behavior among marathon runners.

**Result:**

The environmental factors influencing marathon runners’ sport risk behavior include competition safety climate, sports learning resources, and peers’ risk behavior. Individual factors include achievement motivation, perception of sports risk, and health information seeking behavior. Quantitative research results show that sports learning resources, perception of sports risk, and health information seeking behavior have a significant negative correlation with all dimensions of sport risk behavior. Peers’ risk behavior has a significant positive correlation with all dimensions of sport risk behavior. Competition safety climate has a significant negative correlation with some dimensions. Achievement motivation has a significant positive correlation with some dimensions.

**Discussion:**

Based on the results of this study, it is recommended that competition organizers should pay attention to creating a safe climate during the competition. Relevant social departments, universities, and other organizations should work together to provide sports learning resources for runners and jointly provide an educational environment for marathon runners. These roles should start from the aspects of marathon runners’ exercise motivation, risk perception, and health information seeking skills to help them construct a scientific understanding of fitness.

## Introduction

Marathon is currently a popular sports event worldwide. However, marathon runners need to bear a certain degree of exercise risk during the participation process. Despite the widespread attention and research on safety management issues in recent years, risks such as high incidence of injuries and physical injury accidents still exist in marathon events (Hulme et al., 2017) According to the theory of system security, “human unsafe behavior” is a direct factor in the occurrence of “risk accidents” and should be given attention (Hagger et al., 2022). Regarding this, some researchers have begun to explore the issue of “runner sport risk behavior” in marathon events. On the one hand, it is a survey and research on the current behavior status. Some studies have found that runners themselves have sports risk behaviors such as “not respecting the body’s limits,” “haste to train more” (Saragiotto et al., 2014), and “raining with injuries” (Jelvegård et al., 2016) On the other hand, there is a study on the influencing factors of sport risk behavior among marathon runners. However, these studies currently have limitations. Firstly, most studies have analyzed the impact of “individual factors” on the sport risk behavior of marathon runners, but have not considered environmental and social factor (Zuo et al., 2021). This leads to these literature ultimately proposing the viewpoint of “strengthening runners’ safety awareness through educational intervention,” but it is difficult to answer the question of “how to carry out educational intervention.” Secondly, although some studies consider individual and social factors (Von Rosen et al., 2018), these studies mainly use a single qualitative research method and do not quantitatively verify the results of induction.

Therefore, this study follows the “triangulation” principle of mixed research (using at least three research methods to prove the research results) (Denny and Weckesser, 2019). Firstly, using Bandura’s social cognitive theory as a framework, qualitative research methods are employed to identify the influencing factors of marathon runners’ sport risk behavior, including specific environmental and individual factors. Then, the qualitative research results will be quantitatively validated and supported by relevant literature. Ultimately, provide recommendations for risk prevention and control in marathon events, especially for the safety behavior education of runners.

## Related concepts and conceptual model

### Marathon runner

Currently, many scholars support the use of “running habits” to determine whether an individual is a runner, defining “runner” as “someone who has a habit of running” (Frank et al., 2019; Jiang et al., 2023; Kim et al., 2024).

On the premise of “having a habit.” the concept of “runner” has evolved into diverse subcategories, primarily classified based on participation motivation and goals, running experience, and event types. For instance, recreational runners and competitive runners are subcategories derived from the “participation motivation and goals.” Using “running experience” as a criterion, some scholars further classify runners into novice runners, experienced runners and so on (Schmitz et al., 2014). By event type, categories such as trail runners (Joachim et al., 2024), sprint distance runners (Armento et al., 2023), marathon runners (Li et al., 2025) and so on. Thus, the fundamental distinction between “marathon runners” and other runners lies in their “event type” attribute. Consequently, in this study, “marathon runners” are defined as individuals who have run regularly over the past year and participated in at least one marathon or half-marathon competition within that period (Yao et al., 2021).

### Sport risk behavior

According to the phrase “sport risk behavior,” “sport” serves as the qualifier, specifying the domain in which the behavior occurs (the context of physical activity or exercise). “Risky” functions as the core modifier, revealing the inherent nature of the behavior. “Behavior” is the central term, referring to the specific actions or choices made by individuals (sport participants) during their engagement in sports. Thus, the entire phrase centers on the category of “behavior,” more precisely, actions with a “risky” attribute that occur within the specific domain of “sport.”

Referring to the current research on the concept of “sport risk behavior” (Cobey et al., 2013; Ruedl et al., 2010), we extract the common characteristics of this concept: ① Increasing the likelihood and severity of negative consequences. ② The core of behavior lies in individual’s actions, choices, decisions, or operational patterns. ③ Situational dependence. It specifically refers to risk behaviors that occur in sport activity. ④ The danger is targeted. Specifically, the “risk behavior” in sports situations tends to focus on “physical injury” as its danger. Therefore, the “sport risk behavior” referred to in this study refers to the behavior of marathon runners that may pose a risk to their physical health in the context of sports and exercise.

### Conceptual model-social cognitive theory

Social Cognitive Theory (SCT) was proposed by Bandura based on his reflection on psychodynamics, trait theory, and radical behaviorism (Bandura, 1986). It breaks through the traditional behaviorist “one-way decision” thinking and forms the classic “triadic interaction theory model” to explain the influencing factors of individual social behavior. According to the theoretical model, behavioral factors, individual factors, and environmental factors all interact as determining factors. Although the triadic interaction theory model has been used as a theoretical framework for deductive logic in many studies that focus on the influencing factors of behavior (Chai and Ye, 2024), there is still room for innovation in this theoretical model. Specifically, although it emphasizes the proposition that “environment and individual factors influence individual behavior,” in specific research content, the questions of “what are the specific environmental factors that affect a certain behavior?” and “what are the specific individual factors” still belong to inductive logic. Therefore, the triadic interaction theory model provides a scientifically structured but not entirely restrictive guiding graph for encoding and classification in qualitative research, which conforms to the logical principles of this study. Then, previous research in the field of risk prevention and control for marathon runners has mostly focused on individual factors, which has led to many studies ultimately proposing recommendations that only focus on “help runners construct safety cognition.” But it is difficult to answer the question of “what channels should we use to help runners construct cognition.” This study aims to use SCT to summarize new influencing factors in this field.

## Method

### Qualitative research design

Firstly, semi-structured interviews were used as the primary method for collecting qualitative data, with marathon runners being the main interviewees. The interview content includes: ① Experience of participating in marathon sports; ② Experience of injury or bodily injury accidents during this process; ③ The sport risk behaviors that one has engaged in during the process of participating in a marathon; ④ Inducing/inhibiting the occurrence of these risk behaviors. Based on the definition of the concept of “marathon runner,” the selection criteria for interviewees are: runners who have run regularly in the past year and have participated in at least one marathon or half marathon competition within the same year (Yao et al., 2021).

According to research needs, the researchers conducted a two-stage interview process. The first stage is a pre interview from December 2024 to January 2025, aimed at adjusting the interview outline based on feedback and results from the interview process. The researcher participated in the service work of “Running Hundred Alleys” under NIKE company in Jingan District, Shanghai as a “service provider,” and during this process, met a few marathon runners and established trust with them. The second stage is the formal interview, which will take place from February 2025 to April 2025, using a revised interview outline. Using snowball sampling method, a small group of marathon runners from the social organization “Running Hundred Alleys” were selected as preliminary interviewees. During the research process, the principles of sample adequacy and information saturation in qualitative research were followed, which means that when no new information appears during the interview process, it is considered information saturation. This study interviewed a total of 21 representatives of marathon runners (13 males and 8 females, aged 21–47, with running experience of 1–9 years).

The qualitative research analysis method adopts the thematic analysis, and the Nvivo 12 software is used to encode the interview text. Compared with the grounded theory research method that emphasizes the emergence of concepts from data and the formation of new theories, the thematic analysis method focuses more on identifying and describing patterns (themes) in qualitative data, which is suitable for answering questions about “what,” and is also suitable for qualitative research guided by a certain theoretical framework (Denny and Weckesser, 2019).

### Quantitative research design

Based on the results of qualitative research, retrieve corresponding scales (sport risk behavior and influencing factors), collect quantitative data through questionnaire surveys, and verify the correlation between these influencing factors and the level of sport risk behavior of marathon runners.

The measurement of “sport risk behavior” used “Running Risk Behavior Scale” developed by Hu et al. (2022). This scale is derived from 5 evaluates (training program, physical and mental attention, equipment wearing, competition management, environment selection). After project analysis (PA) (Cronbach’s *α* = 0.853), ultimately 20 items were retained. “Training program” represents the risk behavior of runners in terms of running volume management, training plan, and training program. “Physical and mental attention” includes risk behaviors such as training with injuries and participating in competitions with illnesses. “Equipment wear” refers to the risk behavior of runners who do not pay enough attention to running shoes, protective gear, and other equipment. “Competition management” includes risk behaviors such as pre-race diet and post race recovery for runners. “Environment selection” refers to the risk behavior of runners who lack attention to the traffic safety environment, ground materials, and other environmental factors around them during running.

Based on the results of qualitative research, 6 possible influencing factors were identified (competition safety climate, sports learning resources, peers’ sports risk behavior, motivation, perception of sports risk and health information seeking behavior). The measurement of “competition safety climate” referred to “Safety Scale” in “Perception Scale of Event Service Quality” by Jantori (2023). After PA (Cronbach’s *α* = 0.885), 6 items were retained (example: “The competition I participate in will require me to submit recent medical examination reports or health information”). The measurement of “sports learning resources” referred to “Knowledge Resource Scale” in “Running Related Resource Scale” by de Jonge et al. (2018). After PA (Cronbach’s *α* = 0.900), 7 items were retained (example: “There are running training camps or running groups in my community”). The measurement of “peers’ sports risk behavior” referred to the scales of Cruwys et al. (2021) and combined with the content of runner interviews. After PA (Cronbach’s *α* = 0.824), 4 items were ultimately retained (example: “My friends still insist on completing their scheduled high-intensity training when they are physically fatigued or injured”). The measurement of “achievement motivation” referred to the scale from Markland and Tobin (2004). After PA (Cronbach’s *α* = 0.883), 5 items were ultimately retained (example: “Part of the reason why I persist in training and participating in competitions is to receive praise in my social circle or running teams”). The measurement of “perception of sports risk” referred the “Sports Risk Perception Scale” by Liu et al. (2023). After PA (Cronbach’s *α* = 0.845), 4 items were ultimately retained (example: “I think it is possible for me to face the risk of illness due to improper training”). The measurement of “health information seeking behavior” referred to the scales by Zamani et al. (2014). After PA (Cronbach’s *α* = 0.900), 8 items were retained (example: “I am willing to pay to seek personalized training guidance from a professional running coach”). All scales use a 5-point Likert scale (“1” = complete non conformity, “2” = relatively non conformity, “3” = occasional conformity, “4” = basic conformity, “5” = complete conformity.

Data collection is divided into pre-survey and formal survey. The pre-survey questionnaire will be distributed at the 2025 Lanzhou Marathon and Helong Half Marathon, and the data will be used for project analysis. The formal questionnaire survey will be conducted at the Hangzhou Marathon in 2025, with a total of 514 samples obtained, which is 9.35 times the number of questions (*n* = 55) and meets the requirements of data analysis. The sample information from formal survey is shown in Table 1.

**Table 1:** Sample information for quantitative research.

Variable		Frequency (Percentage)
Gender	Male	311 (60.5%)
	Female	203 (39.5%)
Age	16–20	32 (6.2%)
	21–30	208 (40.5%)
	31–40	141 (27.4%)
	41–50	78 (15.2%)
	51–60	37 (7.2%)
	≥61	18 (3.5%)
Running experience	≤1 year	74 (14.4%)
	2–4 years	262 (51%)
	5–7 years	141 (27.4%)
	≥8 years	37 (7.2%)

Descriptive statistical analysis and Kolmogorov–Smirnov normality test were performed on quantitative data using SPSS 26.0. The mean values of the measurement indicators ranged from 2.19 to 3.89, and the standard deviations ranged from 0.66 to 1.18 (Table 2). The statistics of Kolmogorov–Smirnov test and Shapiro–Wilk test are both significant (*p* < 0.01), indicating that the data does not follow a normal distribution. Therefore, Spearman correlation analysis was chosen to explore the relationship between influencing factors and sport risk behavior of marathon runners. Statistical significance was set at *p* < 0.05.

**Table 2:** Descriptive statistics of variables.

Variable		Mean	SD
Sport risk behavior	Total	2.24	0.66
	Training program	2.23	0.90
	Physical and mental attention	2.22	0.90
	Equipment wearing	2.24	0.96
	Competition management	2.30	0.94
	Environment selection	2.19	1.18
Competition safety climate		3.89	0.87
Sports learning resources		3.78	0.89
Peers’ risk behavior		2.25	0.91
Achievement motivation		2.26	0.96
Perception of sports risk		3.68	0.94
Health information seeking behavior		3.82	0.84

## Result

### Thematic analysis result

Based on the thematic analysis method, the interview text of marathon runners was encoded, and a total of 6 themes and 15 sub-themes were sorted out (Table 3). The following will provide descriptive statements for each theme.

**Table 3:** Thematic analysis results of influencing factors on sport risk behavior of marathon runners.

Theme	Sub-theme	Node	Example Sentence
Competition safety climate	Competition security services	Safety tips in the competition manual	“The participating runners will receive a manual that will remind you of what not to do.”
		Encourage physical examinations	“The competition I participated in this time encouraged us to participate in physical examinations.”
	Obedience of participants	Participants do not pay attention to physical examinations	“Those average level contestants around me usually do not go to get a health report because it’s not mandatory.”
		Participants’ irregular pre-match diet	“The person I met at that time said it was okay to have a drink the day before.”
		Participants’ inappropriate clothing	“Some young people do not wear sportswear during competitions, and there are also those who make it very fancy.”
Sports learning resources	Social group sports learning resources	Coach team	“Our training camp has a team of coaches, some who talk about nutrition, and some who lead training.”
		Running team members share their experiences	“We usually communicate and analyze each other’s movements when running in groups.”
		Running assessment service	“Through this testing service, one can recognize their training issues.”
		Assist in developing training plans and routes	“There will be coaches here who will customize training plans for us.”
		Lack of popularization of running related guidance resources	“Many people do not have access to such good resources.”
	Community sports learning resources	Fitness guidance	“There will be some strength training classes every week that you can sign up for.”
		Scientific fitness lectures and symposiums	“There was once an exchange meeting on sports nutrition held here.”
	Online sports learning resources	WeChat group push	“I often receive content related to running in my WeChat group.”
		Recommended content on social media	“Because I often search for content related to running, clicking ‘recommend’ will list a lot for me.”
Peers’ sport risk behavior	Peers’ behavioral outcomes	Everyone around me has “nothing wrong”	“I do not think anyone around me has done anything big, so this should not be a problem.”
	Peers’ behavior demonstration	Peers training with injuries	“Most of the team members are injured and will continue to train.”
		Peers dress casually	“I do not think they are wearing professional sportswear either, just wear it casually.”
		Peers’ casual pre match preparation	“Those seniors were still practicing the day before the competition, let us follow them.”
Motivation	Utilitarian-oriented	Declaration of pacer requires proof of performance	“Because the declaration of a speedometer required a full marathon score, I also applied for a full marathon score at that time.”
		Competing for bonuses	“We all wanted to compete for the prize money in that team competition.”
	Group identification	The collective honor of the running team/group	“Although it hurts, it cannot hold everyone back.”
		Runner’s spirit	“If you cannot reach this amount, you cannot be considered a ‘runner’.”
	Breakthrough	Create the best achievements	“At that time, I was wondering if I could break through the limit again.”
		Try	“I have been practicing for a long time and I do not want to waste this opportunity, but that fainting was really scary.”
	Health and leisure	“Health first”	“At my age, good health is enough.”
		Not caring about others’ grades	“They will achieve good results, let them go, do not compare with others.”
Perception of sports risk	Self perception of bodily signals	Physical reactions during exercise	“If the pace is too fast, my body will have some reactions. At this time, I will adjust it.”
		Pain	“Now whenever it hurts, I feel like there might be a problem with my training.”
		Injury	“After that injury, I knew I could not do it again.”
		Capable of withstanding	“If you think you can hold on, then keep running.”
	Risk perception of events	Understanding the risks of marathon projects	“After that competition, I knew this project could not be treated arbitrarily.”
		Understanding of sports risks	“As long as it’s sports, there is definitely a possibility of injury, so it’s important to take precautions.”
Health information seeking behavior	Proactively search for information	Search for knowledge online	“Now the Internet is very developed. I will search for some relevant content online.”
		Consultation	“I did not have to go to the hospital for a check-up that time, otherwise I might have continued practicing like this. Since then, I still trust doctors more.”
		Read theoretical books	“Part of the knowledge is read from that ‘Running Bible’.”
	Information resolution ability	Difficult to distinguish authenticity	“The things online are really ‘miscellaneous’, and it’s relatively rare to find truly effective ones.”
		Inappropriate methods adopted	“Just follow the content online and find it increasingly painful.”

### Competition safety climate

The concept of a safe climate originated in the field of enterprise management, first proposed by Zohar (1980) in 1980, referring to “employees’ perception of a risky environment.” At present, with the diffusion of the “safety climate” theory in different fields, concepts such as enterprise safety climate (Sullman et al., 2017) and road safety climate (Doncel et al., 2023) have gradually extended. The concept of safety climate in different contexts has the following commonalities: Firstly, it is perceptible. Individuals in a specific context can “feel” the priority of safety in that context through daily observation and experience. Secondly, it’s multidimensionality. The safety climate is not a single dimensional concept, it includes both the perception of the management’s emphasis and commitment to safety, as well as the perception of the “support response” of relevant safety systems in the practical process. Through thematic analysis, this study first extracted 5 nodes closely related to the perception of event safety climate. Among them, “safety tips in the competition manual” and “encouraging physical examinations” are categorized as sub-themes “competition security services,” representing marathon runners’ perception of the services provided by the competition organizers for safe participation. This sub-theme directly corresponds to the “what the organizers have done,” which is in line with the “management commitment and system” dimension of the safety climate. Secondly, “participants do not pay attention to physical examinations,” “participants’ irregular pre-match diet,” and “participants’ inappropriate clothing” are categorized as sub-themes “obedience of participants,” representing the respondents’ perception of the degree to which participants comply with the safety regulations related to the competition. This sub-theme aligns with the dimension of “support response” in the theory of “safety climate.” These two sub-themes align with the important dimensions of safety climate theory, namely the importance of management and the response of participants. Therefore, placing “safety climate” in “competition” and naming this theme “event safety climate” represents runners’ perception of the overall environment and priority of event safety created by the event organizers in the context of marathon events, as well as participants’ support for safety systems. Comparing the characteristics of the two, it can be found that the former is more direct and mandatory, while the latter is relatively indirect.

### Sports learning resources

The American Association for Educational Communication and Technology (AECT) stated in 1994 that “learning resources” include “resources, environments, and tools that support learning,” and are the sum of all human and non-human, material and non-material elements that support learners in achieving learning goals. The core question answered by the concept of “learning resources” is “through which channels and carriers can learners acquire knowledge and skills to achieve learning goals.” Through thematic analysis, this study identified 9 nodes closely related to the channels through which runners obtain scientific fitness knowledge and running training plans. “Social group sports learning resources” refers to whether runners have resources closely related to running activities such as running teams and training camps. Compared to the former, “community sports learning resources” is more universal. The emergence of the sub-theme “online sports learning resources” indicates that some runners’ learning channels have exceeded the former’s “presence” requirements. Having/lacking these channels for acquiring knowledge is considered by the runners in this study as a factor that inhibits/generates their sport risk behavior. Because the content of these nodes is related to the “sports” context, this study named the topic “sports learning resources,” representing the channels through which marathon runners can obtain knowledge related to scientific fitness and running (such as scientific training plans, sports nutrition knowledge, and sports injury management knowledge) in their fitness experiences.

### Peers’ sport risk behavior

Through thematic analysis, this study identified four nodes closely related to peers’ behavior and outcomes. The sub theme “peers’ behavioral outcomes” refers to the perception formed by the interviewee that their peers engage in a certain sports risk behavior (such as playing with injuries) without experiencing serious sports injuries or health accidents. By observing the “results brought by the behavior,” the interviewee forms a cognition of the danger of the behavior. “Peers’ behavior demonstration” refers to the specific behaviors observed by the interviewee’s peers. Based on the above content, it can be seen that the sources of influence for the two sub-themes referring to sports risk behavior are both “peers around runners.” Therefore, this theme is named “Peer Sports Risk Behavior.”

It is worth noting that the “Peers’ casual pre-match preparation” node in this theme is somewhat related to the “obedience of participants” in the “competition safety climate,” but there is also a distinction. The concept of “peers” identified in this study refers to the friends and teammates that marathon runners are familiar with during their daily training and participation in the event. Compared to the observed participants, the interaction of “peers” is closer to runners.

### Motivation

Sports motivation refers to the internal psychological drive that drives individuals to participate in sports activities, which is the intrinsic reason for stimulating, maintaining, and regulating sports behavior (Rogowska and Morouço, 2024). Through thematic analysis of interview results, 8 nodes related to sports motivation were extracted and classified into 4 sub-themes based on the type of motivation. In the interviewee’s statement, these factors drive them to engage in/avoid some risky sports behaviors. These sub-themes reflect the guiding role of different motivations on the behavior of the respondents in their running career. Therefore, these sub-themes are condensed into the theme of ‘motivation’.” “Utilitarian-oriented,” “group identification” and “breakthrough” are closely related to some risk behaviors of the respondents in the study, especially behaviors such as “participating in competitions with injuries” and “training with injuries.” All the three sub-theme belong to the motivation of achievement. On the contrary, the motivation of “health and leisure” seems to suppress these behaviors of runners.

### Perception of sports risk

Perception of sports risk refers to the subjective assessment and judgment process of potential dangers, potential injuries, and the severity of consequences that individuals may experience while participating in sports activities (Martha et al., 2009). It is not an objective risk itself, but rather an individual’s understanding and interpretation of risk factors based on their own cognition, experience, and external environmental information (Yamaguchi and Ito, 2021). This study extracted six nodes related to risk perception through thematic analysis of interview results, and classified them into 2 sub-themes based on the perceived objects. The first is the risk perception of runners towards their own bodies, and the second is the risk perception towards marathon events. From this, we can find that the object of runners’ perception of sports risks not only includes “self” (correctness of their own behavior and physical state), but also includes “non self” (risk perception of events).

### Health information seeking behavior

Health information seeking behavior is an important concept in the field of health behavior research. Through thematic analysis, this study identified 5 nodes related to runners’ behavior of seeking health and scientific fitness related information. According to the interviewee’s statement, these nodes have influenced their understanding of health knowledge, fitness knowledge, and their own condition, thereby affecting their sport risk behavior. This theme reflects the behavioral process of respondents actively acquiring information and knowledge related to exercise and health, and identifying them. Therefore, this study summarizes the above sub-themes as the theme of “health information seeking behavior.” “Proactive search for information” and “information resolution ability” jointly affect the risk behavior of runners. If runners have the former but not the latter, it can also lead to them being misled, resulting in unscientific training behaviors and injury management behaviors.

It is worth noting that there is a certain correlation between this theme and “sports learning resources,” both focusing on how marathon runners “obtain knowledge and information from the environment.” However, the difference is that “health information seeking behavior” focuses on the runners’ own active behavior, while “sports learning resources” focus on the accessibility and accessibility of knowledge and information in the environment.

### Quantitative verification results

The quantitative results are presented in Table 4. The competition safety climate is significantly negatively correlated with some dimensions of sports risk behavior (*p* < 0.01). Specifically, there is a significant negative correlation between “environment selection” (*r* = −0.223, *p* < 0.01), “equipment wear” (*r* = −0.211, *p* < 0.01), and “competition management” (*r* = −0.157, *p* < 0.01), indicating that the better the safety climate created by the event organizers (such as safety tips), the lower the level of risk behavior of runners in daily training environment selection, equipment wear, and self-management during the competition process. However, this element was not significantly correlated with the dimensions of “training program” (*r* = −0.057, *p* > 0.05) and “physical and mental attention” (*r* = 0.069, *p* > 0.05).

**Table 4:** Spearman correlation coefficient between variables.

Variable	SRB (TP)	SRB (P&M)	SRB (EW)	SRB (CM)	SRB (ES)
Competition safety climate	−0.057	0.069	−0.211^**^	−0.157^**^	−0.223^**^
Sports learning resources	−0.130^**^	−0.118^**^	−0.223^**^	−0.219^**^	−0.177^**^
Peers’ risk behavior	0.141^**^	0.167^**^	0.210^**^	0.135^**^	0.197^**^
Achievement motivation	0.006	0.093^*^	0.070	0.049	0.017
Perception of sports risk	−0.254^**^	−0.200^**^	−0.292^**^	−0.194^**^	−0.248^**^
Health information seeking behavior	−0.176^**^	−0.113^*^	−0.131^**^	−0.166^**^	−0.084

SRB, Sport risk behavior; TP, Training program; P&M, Physical and mental attention; EW, Equipment wearing; CM, Competition management; ES, Environment selection. ^*^(*p* < 0.05); ^**^(*p* < 0.01).

There is a significant negative correlation between sports learning resources and all dimensions of sports risk behavior. Among them, the negative correlation strength with the dimensions of “equipment wear” (*r* = −0.223, *p* < 0.01) and “competition management” (*r* = −0.219, *p* < 0.01) is relatively high, indicating that the richer the learning resources obtained by runners, such as sports knowledge and training guidance, the more they pay attention to the correct use of equipment and safe behavior during the competition process. The negative correlation with the dimensions of “environment selection” (*r* = −0.177, *p* < 0.01), “training program” (*r* = −0.130, *p* < 0.01) and “physical and mental attention” (*r* = −0.118, *p* < 0.01) is also significant, indicating that sufficient sports learning resources can prompt runners to pay attention to the rationality of their training plan, focus on their physical and mental state, and choose a safe running environment.

Peers’ sport risk behavior is significantly positively correlated with all dimensions of sport risk behavior. Among them, the positive correlation with the dimensions of “physical and mental attention” (*r* = 0.167, *p* < 0.01) and “environment selection” (*r* = 0.197, *p* < 0.01) is the most prominent. The positive correlation with the dimensions of “equipment wear” (*r* = 0.210, *p* < 0.01), “training program” (*r* = 0.141, *p* < 0.01) and “competition management” (*r* = 0.135, *p* < 0.01) also reached a statistically significant level, reflecting the “demonstration effect” of peer behavior on runners’ risk decision-making.

Achievement motivation is only significantly positively correlated with the “physical and mental attention” dimension (*r* = 0.093, *p* < 0.05), and has no significant correlation with other dimensions, indicating that the stronger the motivation of runners to pursue achievements such as grades and social recognition, the weaker their physical and mental attention towards themselves.

The perception of sport risk is also significantly negatively correlated with all dimensions of marathon runners’ sport risk behavior. Specifically, the absolute correlation coefficient between sports risk perception and the “equipment wear” dimension is the highest (*r* = −0.292, *p* < 0.01), indicating that the higher the level of perception of sports risk by runners, the more inclined they are to wear appropriate sports equipment in a standardized manner to avoid risks. Secondly, there is a significant negative correlation with the dimensions of “environment selection” (*r* = −0.248, *p* < 0.01) and “training program” (*r* = −0.254, *p* < 0.01), indicating that runners with strong risk perception pay more attention to safety and scientificity when choosing training environments and developing training plans. There is also a significant negative correlation between risk perception and the dimensions of “physical and mental attention” (*r* = −0.200, *p* < 0.01) and “competition management” (*r* = −0.194, *p* < 0.01), which confirms the positive guiding effect of risk perception on runners to pay attention to their own physical and mental state and regulate their own management.

There is a significant negative correlation between seeking health information and the four dimensions of sport risk behavior. Specifically, there is a significant negative correlation between the dimensions of “training program” (*r* = −0.176, *p* < 0.01), “physical and mental attention” (*r* = −0.113, *p* < 0.05), “equipment wear” (*r* = −0.131, *p* < 0.05), and “competition management” (*r* = −0.166, *p* < 0.01), indicating that the stronger the behavior pattern of runners actively seeking health knowledge, the higher their attention to training scientificity and physical and mental health, and the higher their level of equipment selection and safety behavior during running and competition.

## Discussion

This study first conducted a thematic analysis to summarize the possible influencing factors that induce sport risk behavior among marathon runners. The environmental factors mainly include the safety climate of the event, sports learning resources, and peers’ sport risk behavior. Individual factors include exercise motivation, perception of sport risk and health information seeking behavior. Subsequently, the quantitative analysis results confirmed the relationship between these factors and the sport risk behavior of marathon runners.

The perceived level of safety climate in marathon runners is significantly negatively correlated with their level of sport risk behavior in the dimensions of environment selection, equipment wear, and competition management. At present, the relationship between safety climate and individual risk behavior has been confirmed in the field of sports (Finch et al., 2004) or other safety science fields (Hope et al., 2010; Seo et al., 2015). It is speculated that on the one hand, this may be due to the mandatory impact of “explicit regulation” on the behavior of runners (through clear systems and prompts such as competition manuals and medical advice, directly informing runners of what constitutes risk behavior), and on the other hand, it may be a common result of “implicit guidance” (runners internalize this value orientation when they perceive that race participants prioritize safety highly).

There is a significant negative correlation between sports learning resources and all dimensions of runners’ sport risk behavior. In this study, “sports learning resources” are the foundation for marathon runners to construct scientific training cognition and form correct risk judgment abilities. The relationship between learning resources and risk behavior has been validated in research on risk behavior in various fields (Vredenburgh, 2002). This relationship can be explained by the “environmental constraints” (Bandura, 1986) of SCT. Bandura emphasizes that when environmental conditions impose strong constraints on behavior, the environment emerges as the dominant determining factor. The scarcity of learning resources (such as lack of access to running technology guidance services, and absence of scientific fitness guidance public welfare courses in the community) may make it difficult for runners to construct scientific fitness knowledge or have a clear understanding of their own reality (such as whether their running posture is correct and whether their dietary patterns are healthy). On the contrary, runners with abundant resources can use assessment services to understand their real situation, and learn scientific fitness knowledge through course learning and training camps. It is speculated that these resources indirectly affect the behavior of runners under the inherent logical relationship of “cognition behavior.”

Peer sport risk behavior shows a significant positive correlation with all dimensions of risk behavior. In this study, “peers’ sport risk behavior” refers to the behavior observed by marathon runners within their social circle (such as running teams, training partners, etc.) that deviates from scientific fitness principles and may lead to physical health hazards. It is a social environmental factor that is carried by people. According to SCT, people can construct their cognition of a certain behavior through observational learning, and the behavior and outcomes of the demonstrator will participate in this process. The “significance” and “observability” of the demonstrated activity are important factors that affect the “attention process” (Bandura, 1986). Companions, as the objects that marathon runners are more likely to pay attention to, should naturally become the priority “demonstration activity” and be observed during the attention process stage. On the other hand, Bandura also pointed out that the attention process is influenced by the outcomes of others’ experiences and the outcomes of individuals’ direct experiences. The result of “no incident” can be a powerful “alternative experience,” sending a signal to the observer that “this behavior is safe,” thereby strengthening their motivation to imitate. The relationship between these two has also been validated in research in sports (Von Rosen et al., 2018) and other fields (Yusliza et al., 2017).

Among individual factors, the perception of sport risk is a protective factor, which is significantly negatively correlated with the sport risk behavior dimensions of most marathon runners in this study. This result supports the viewpoint of Mohamed (Kapadia, 2017). From other fields, there are also studies confirming the potential impact of an individual’s perception of risk on risk behavior (Boua et al., 2022).

The process by which health information seeking behavior affects runners’ sport risk behavior may be explained by the “non simultaneity” principle (Bandura and Barab, 1971) in SCT. Although “behavior” was used as an outcome measure in this study, it is not the endpoint of the SCT model and also constitutes one of the factors that affect the environment and individuals. In addition to influencing human behavior, the environment is also influenced by behavior. When individuals seek a certain environment autonomously and actively, new environmental factors are generated, and then enter the next process of interactive influence, presenting an overall development process with vector characteristics. In the field of education, the following example can be given: when an individual remains silent about a certain learning resource, it is difficult to explore the related learning environment and naturally form relevant cognition. On the contrary, if it maintains the goal and psychology of active learning, and develops proactive behavior of seeking learning resources, it may change the relevant learning environment, which is conducive to shaping corresponding cognition. The relationship between health information seeking behavior and risk behavior has been validated in existing research (Weaver III et al., 2010).

Motivation is one of the influencing factors of marathon runners’ sport risk behavior. The thematic analysis results of this study show that runners who prioritize performance and social identity as their main motivations often exhibit behavior patterns of overtraining and injury training. The results of quantitative analysis also show a significant positive correlation between achievement motivation and runners’ sport risk behavior in the dimensions of “physical and mental attention.” This can be explained by the “self-regulation mechanism” (Bandura, 1986) in SCT. The self-regulation mechanism refers to the establishment of inner evaluation standards for a certain behavior or thing through learning, which regulates people’s behavior. If marathon runners evaluate their behavior based on “achievement” rather than “health,” they may exhibit risk behaviors such as training with injuries and neglecting physical and mental attention. The relationship between this element and sport risk behavior has been validated in other studies (Chan and Hagger, 2012; Perera et al., 2019).

The theoretical contribution of this study lies in: on the one hand, introducing the situational elements and sports background of “marathon” into SCT, enriching the empirical research cases of SCT. On the other hand, this study identified influencing factors that have not been identified in previous research in this field. For example, environmental factors related to education such as “sport learning resources.” And we have demonstrated that individual factors that influence runners’ risk behavior not only include psychological factors, but a certain behavior may also indirectly affect runners’ sport risk behavior by changing the environment (health information seeking behavior may affect runners’ learning resources and thus affect their cognition and risk behavior).

In terms of practical contribution, the findings of this study provide a certain direction for intervening in the risk behavior of marathon athletes. The key individual factors identified in this study (motivation, perception of sports risks, and health information seeking behavior) may become the “content” of marathon runner education, while environmental factors (competition safety climate, sports learning resources, and peers’ sports risk behavior) may become the “form” of educational intervention. Specifically, firstly, the organizers of the competition should strengthen safety reminders for participants (such as requiring participants to undergo physical examinations and promoting scientific pre-competition dietary knowledge to participants through social media). In this study, “sports learning resources” were identified as an important factor affecting runners’ cognitive construction. “Lack of resources” can lead to confusion about whether their training plan is reasonable, whether their gait is scientific, and whether their way of handling injuries is appropriate. Therefore, the accessibility of resources such as sports guidance services is worth paying attention to. This may be achieved through the cooperation of relevant social departments, universities, enterprises, and other roles with teaching resources. Meanwhile, this study found that “media” is also one of the ways for marathon runners to acquire scientific fitness knowledge. Therefore, perhaps educational interventions through media dissemination can help runners improve their awareness of sports risks and thereby suppress their sports risk behaviors. This viewpoint has been validated in the research of others (Hespanhol et al., 2018). The peers’ effect is also a noteworthy issue, and it is recommended that other researchers take “social running groups” as the research object to conduct in-depth research on the collective behavior patterns of this community. Finally, the results of this study suggest that we may also intervene in the sport risk behavior of marathon runners by correcting exercise motivation, popularizing knowledge of sport risks, and enhancing health information seeking skills.

## Limitation

The study has several limitations. First, the sample was exclusively composed of Chinese marathon runners, which may limit the generalizability of the results to other cultural or regional contexts. Second, although this study summarizes multiple influencing factors of marathon runners’ sport risk behavior, using different theoretical frameworks may identify more influencing factors.

## Data Availability

The raw data supporting the conclusions of this article will be made available by the authors, without undue reservation.
